# In Vitro and In Vivo Studies of Antibacterial Coatings on Titanium Alloy Implants for Veterinary Application

**DOI:** 10.3390/ijms24098114

**Published:** 2023-04-30

**Authors:** Magdalena Ziąbka, Katarzyna Matysiak, Katarzyna Cholewa-Kowalska, Agnieszka Kyzioł, Aleksandra Królicka, Rafał Sapierzyński, Monika Januchta-Kurmin, Igor Bissenik

**Affiliations:** 1 Faculty of Materials Science and Ceramics, Department of Ceramics and Refractories, AGH University of Science and Technology, 30-059 Krakow, Poland; 2 Faculty of Materials Science and Ceramics, Department of Glass Technology and Amorphous Coatings, AGH University of Science and Technology, 30-059 Krakow, Poland; 3 Faculty of Chemistry, Jagiellonian University, 30-387 Krakow, Poland; 4 Laboratory of Biologically Active Compounds, Intercollegiate Faculty of Biotechnology UG-MUG, University of Gdansk, 80-307 Gdansk, Poland; 5 Department of Pathology and Veterinary Diagnostic, Institute of Veterinary Medicine, Warsaw University of Life Sciences-SGGW, 02-776 Warsaw, Poland; 6 Veterinary Clinic “Pulawska”, 02-844 Warsaw, Poland

**Keywords:** bactericidal efficacy, composite hybrid layers, cytotoxicity, nanoparticles, tibial plateau leveling osteotomy

## Abstract

The aim of this work was the evaluation of biological properties of hybrid coatings modified with Ag, Cu, and Zn nanoparticles (NPs) applied on TPLO medical implants by the sol-gel process. The implant coatings enriched with various concentrations of metallic NPs were investigated in the in vitro bactericidal efficacy tests against Gram+ and Gram- bacteria and pathogenic yeast. Next, the designed materials were tested on human osteosarcoma cell lines. The cells adhesion, proliferation, viability, and differentiation were investigated. The cell growth wasevaluated using SEM, and the metallic ion release was measured. The results revealed that the NPs concentration in the hybrid layers decreased with the incubation time. In the last stage, the implants were tested in vivo on six canine patients. Three months after the operation, the radiological evaluation of the performed anastomosis was carried out as well as the histopathological evaluation of tissue regeneration. The strongest bactericidal efficacy was observed for the layers containing AgNPs. Along with an increased concentration of metallic additives, a growing toxic effect was clearly observed. The most pronounced toxic effect was especially evident with the AgNPs concentration exceeding 1 mol %. In all the operated patients, no deviations were found during the follow-up examinations in the postoperative period. The low dose of AgNPs in the hybrid layer facilitated the tissue healing process. It was proven that silver nanoparticles may accelerate the bone healing process. The correct tissue reparation was observed.

## 1. Introduction

The tibial plateau leveling osteotomy (TPLO) has become a common method to treat the cranial cruciate ligament rupture in dogs. Several types of implant systems are used to stabilize the osteotomy of the tibia during the TPLO [1]. Most of them differ in fixation techniques with various types of plates applied, e.g., nonlocking, locking, double plating, and contoured locking compression plates [2]. The implant efficacy and the surgery complication rate determine the choice of the appropriate set. The most commonly used systems are fabricated of titanium alloys and 316 L stainless steel [3], and also cobalt chromium molybdenum, but the latter ones are rarely used in veterinary medicine due to complications including loosening and tissue necrosis [4]. However, steel implants frequently induce the focal osteolysis adjacent to the plate and the implant corrosion with the particulate matter present in the tissues. Furthermore, due to their toxic effects, the corrosion products may negatively affect the osteoblast proliferation and differentiation. They may also accumulate, leading to adverse tissue reactions such as: acute inflammation, granulation tissue, collagen formation, and tissue necrosis [5]. Yet another complication connected with the TPLO metallic implants is the surgical site infection (SSI) caused by bacterial contamination [6,7,8]. The SSI not only has a negative impact on the patient’s well-being, but it often necessitates the antibiotics administration or an additional anesthetic episode of the surgical implant removal [9]. Prolonged surgical infections and antibiotic therapies significantly influence the hospitalization length and its costs; they are also painful and stressful for patients. In view of the above factors and the growing microorganism resistance to antibiotics, scientists are attempting to implant surface modifications, their functionalization, and development of antibacterial coatings. Introduction of inorganic antibacterial agents, e.g., silver, copper, and zinc, onto the surface of titanium substrate results in obtaining a sufficient antibacterial effect [10]. There are various methods to improve the physicochemical and biological properties of implant surfaces already described in the literature. The most popular techniques include: ion implementation [11], in situ reduction [12], electrophoresis [13], plasma electrolytic oxidation [14], sol-gel [15], sputtering [16], or plasma spray [17]. The sol-gel method is relatively convenient, and it allows excellent control of the coating composition. On one hand, it protects the surface against scratches and corrosion, on the other hand, it exhibits antibacterial properties without hampering the tissue integration. Due to this, thin films of virtually any shape can be formed on titanium substrates. The hybrid coating provides good adhesion to metallic surfaces, and the nanoparticles of silver, copper, or zinc formed in the coating boost the surface properties, e.g., bactericidal action. Additionally, numerous compounds, e.g., antibiotics or active metallic ions, can be incorporated into the coatings and locally delivered at a controlled rate [18,19,20]. Moreover, the sol-gel technique is low-cost and versatile. Via this method, it is possible to coat elements of geometrically complex shapes, in contrast to other methods which are limited to covering relatively simple shapes.

The Ti6Al4V alloy implants were selected for the study due to their optimal mechanical properties, very good corrosion resistance, and an exceptional degree of biocompatibility [21]. However, they also display relatively poor tribological properties [22]. Therefore, they require surface treatments to effectively improve their surface strength and prevent bacterial infection associated with the implantation procedure. An aluminum additive reduces the specific gravity of titanium and strengthens a solid solution, while vanadium stabilizes the β phase. On the other hand, the vanadium release of vanadium ions into the surrounding tissues is problematic, as it causes cytotoxic reactions and consequently, neurogenic and carcinogenic disorders [23,24].

In our previous work, novel hybrid coatings based on organically modified silica and titanium precursors enriched with silver, copper, or zinc nanoparticles were prepared and comprehensively evaluated in terms of the structural and surface properties [25]. The results confirmed that in the composite layer obtained by the sol-gel method, nanoparticles in the organosilicon matrix were formed during thermal treatment. Additionally, the surface properties (wettability, surface free energy, and roughness) depended on the type and size of NPs, i.e., the best homogeneity and lower surface free energy (SFE) were noticed for the layers with silver nanoparticles. All the coatings ensured the smoother alloy surface and protected it against surgical scratches. In our current study we used commercially available Ti6Al4V alloy, commonly used for TPLO implants, where good mechanical characteristics and biocompatibility are essential. The novelty of the study is the implementation of the sol-gel technique to protect the modified veterinary implants against corrosion and scratching. The new hybrid layers also ensure the antibacterial action and biocompatibility. The research presented here is a continuation of the study with a particular focus on antibacterial properties, leading to the selection of the optimal concentration and type of nanoparticles in terms of cellular response. Furthermore, the influence of AgNPs on the tissue regeneration process was thoroughly investigated in vivo.

The studies described and discussed in the article prove the hypothesis that it is possible to create an organic–inorganic antibacterial layer on veterinary implants (TPLO) via the sol-gel method, while limiting the cytotoxic effect of AgNPs, CuNPs, and ZnNPs nanoparticles.

## 2. Results

### 2.1. In Vitro Examinations

In this work the medical implants (TPLO system fabricated of TiAlV alloy) intended for long bone anastomoses in animals were covered with hybrid layers containing zinc, copper, and silver nanoparticles obtained via the sol-gel process through a dip-coating method.

Based on the biocidal efficacy results, it was concluded that the antimicrobial effectiveness increased with the increasing content of metallic nanoparticles. Silver nanoparticles indicated the strongest efficacy against both Gram-positive bacteria (*S. aureus*, *E. faecalis*) and Gram-negative (*E. coli*, *P. aeruginosa*) bacteria (Table 1) in comparison with the control samples (Ti and hybrid).

A total of 100% of bactericidal efficacy was observed for all the AgNPs contents in the layers. Even in the 0.5% Ag layer with 99.9% bactericidal activity against *S. aureus*, a 3-logarithm decrease in the number of bacteria was observed compared with the control (Ti and hybrid).

Among the other tested layers containing zinc and copper nanoparticles, the 5% copper ones had the highest antimicrobial activity. In this sample, the bactericidal effect (3-log decrease) was observed for three out of four tested microorganisms. Only *S. aureus* showed a decrease of 1-logarithm. The inverse relationship was observed in the case of the zinc nanoparticle layers where a 5% zinc content caused a bactericidal effect against *S. aureus*. In the case of fungicidal activity, the 5% AgNPs layer was characterized by the highest effectiveness from all the silver concentrations. In this case, the number of fungal colonies (CFU) decreased by 1-logarithm. For the 5% copper samples, a decrease of 1-logarithm was observed while a 2-logarithm decrease was observed for the 5% ZnNPs samples, compared with the control (Ti and hybrid). It was the highest observed fungicidal activity of all the tested layers.

**Figure 1:**
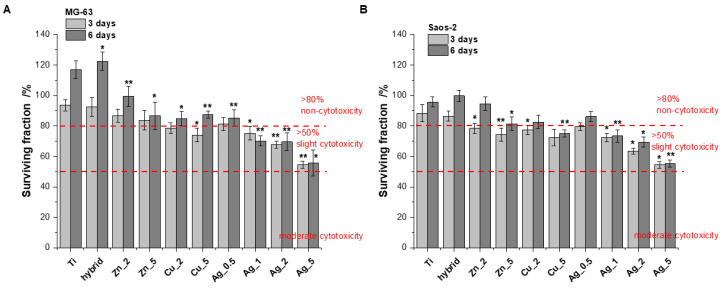
**Figure 1**. The proliferation of MG-63 (**A**) and Saos-2 (**B**) cells on the modified and unmodified TiAlV surfaces evaluated by Alamar Blue assay conducted at time points of 3 and 6 days (data based on triplicate samples of each substrate ± SD). Stars indicate the data of the level of significance for cells treated with various modifications versus cells treated with unmodified TiAlV for 3 days and 6 days, respectively (Student’s *t*-test, * *p* ≤ 0.05, ** *p* ≤ 0.005).

The biological response to all the Ti alloy surfaces was evaluated using human bone-derived cell lines (MG-63 and Saos-2). Due to their capability of rapid bone healing, osteosarcoma cells are widely used in bone cell differentiation, proliferation, and metabolism research [26]. In our study the cell proliferation was measured using the Alamar Blue assay conducted after 3 and 6 days. The tests were performed on the unmodified surfaces (TiAlV) and the modified ones (TiAlV/h, and TiAlV/h) containing Ag_0.5, Ag_1, Ag_2, Ag_5, Cu_2, Cu_5, Zn_2, and Zn_5. The TiAlV surfaces supported the steady proliferation process over the 5-day incubation with human osteoblast-like (osteosarcoma) cell lines. The cell attachment and proliferation were quantitatively monitored via the conventional optical microscopy, which revealed no morphological changes. The cytotoxicity results, expressed as a surviving fraction of cells after culturing on the investigated TiAlV substrates, are presented in Figure 1.

The MG-63 and Saos-2 cell proliferation on the investigated surfaces of modified Ti alloy substrates suggests no obvious cytotoxicity (relative proliferation rate >80%) [27,28] for all specimens apart from Cu_5, Ag_1, Ag_2, and Ag_5 (after 6 days of incubation). Importantly, none of modifications revealed the moderated toxicity, i.e., the surviving cell fraction below 50%, within the time increase. However, all the substrates showed a clear dependence: an increasing concentration of metallic additives used for the surface modification meant a growing toxic effect. This phenomenon was especially evident with Ag for which the toxic effects were most pronounced.

**Figure 2:**
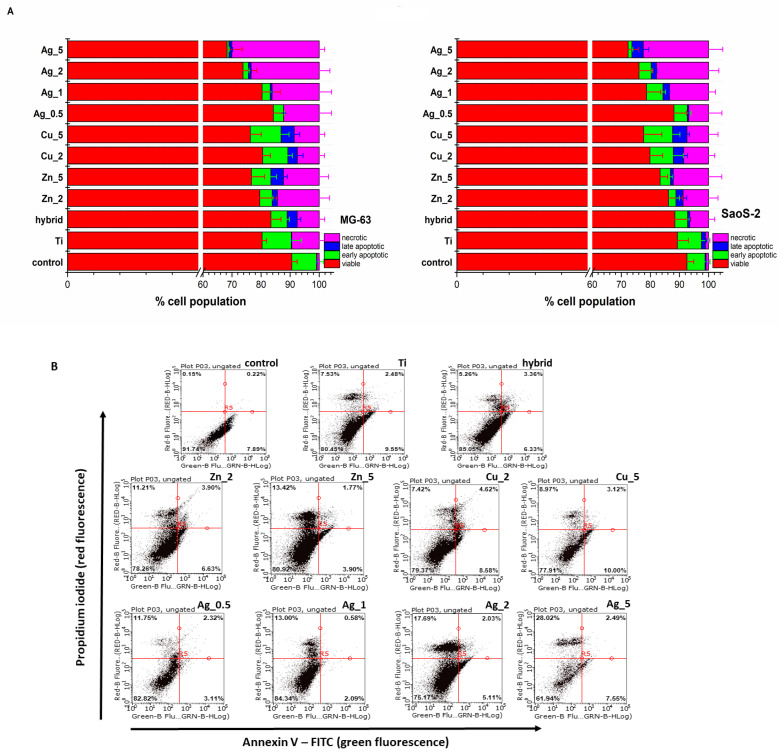
(**A**) The MG-63 and SaoS-2 cells death modes induced by the unmodified and modified Ti substrate surface determined with the Annexin V-FITC/propidium iodide (PI) double staining assay (Annexin V-FITC: green fluorescence; PI: red fluorescence) was used to detect the phosphatidylserine externalization in apoptosis and analyze the membrane integrity, respectively. FACS quantified apoptosis and necrosis after Annexin V-FITC and PI labelling. The relevant cells grown under the same experimental culture conditions on polystyrene plates were treated as control. (**B**) The representative flow cytometry dot plots reveal the MG-63 cell death modes induced by the unmodified (Ti alloy) and modified Ti alloy surfaces (Hybrid, Zn_2, Zn_5, Cu_2, Cu_5, Ag_0.5, Ag_1, Ag_2, Ag_5). The Annexin V-FITC/propidium iodide (PI) double staining assay (Annexin V-FITC: green fluorescence; PI: red fluorescence) was used to detect the phosphatidylserine externalization in apoptosis and determine the membrane integrity, respectively.

The flow cytometry analysis of cell death modes confirmed no significant toxicity for the Ti substrates. The percentages of the population of live cells and those undergoing particular types of cell death (apoptosis and necrosis) are presented in Figure 2.

Most TiAlV substrates exhibited biocompatibility with a population of living cells exceeding 70%. The highest toxicity was observed for Ag_5 with the number of dead cells (apoptotic and necrotic) exceeding 30% in the case of MG-63 cells (Figure 2B). Importantly, having analyzed the cell death type for the Zn and Ag additions, an increasing prevalence of necrosis over apoptosis was noticed, while for the Cu modifications this tendency was not observed; therefore, one might conclude that necrotic damages to the cell membrane caused by the ion release from the outer surface layers are not so significant.

**Figure 3:**
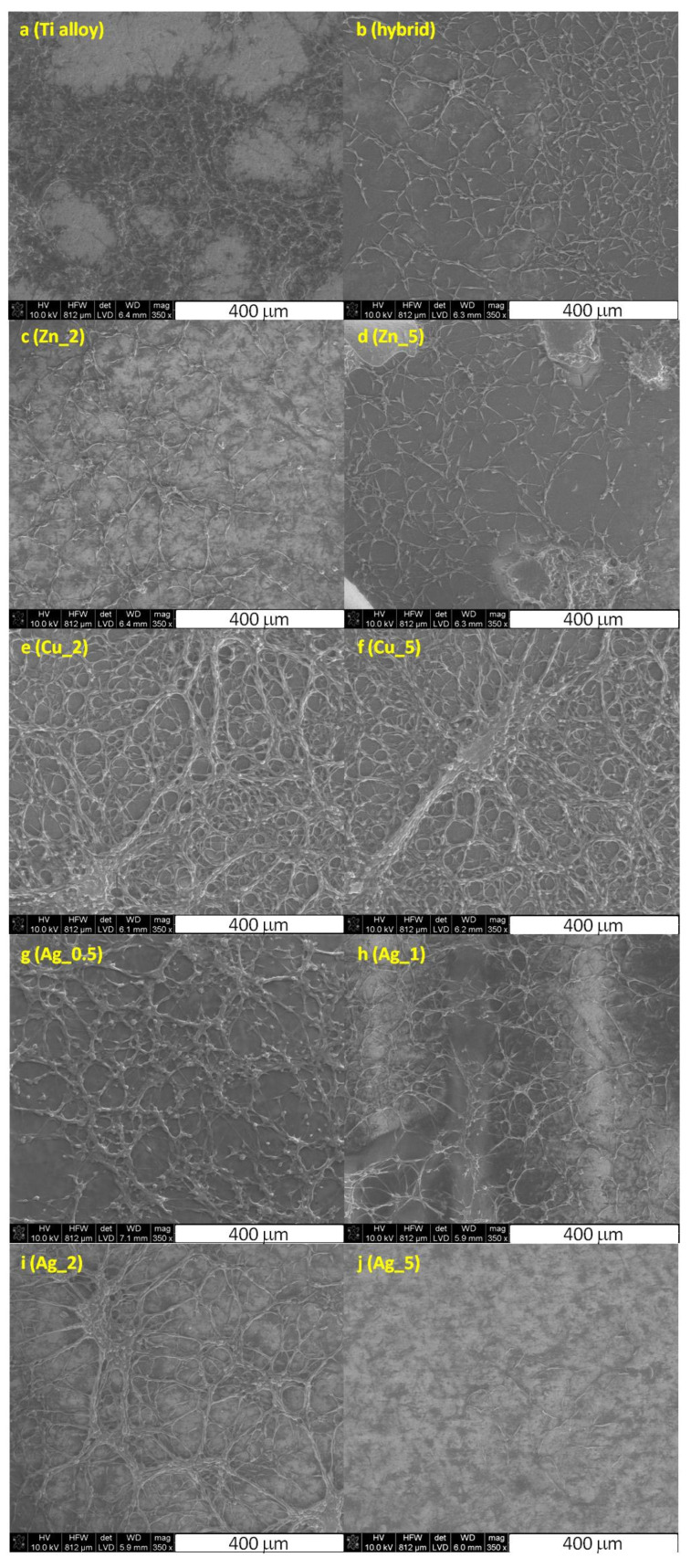
The SEM images of the fixed MG-63 cells cultured on the unmodified Ti alloy (**a**), Ti alloy with hybrid layer (**b**), and modified with nanoparticles of Zn_2 (**c**), Zn_5 (**d**), Cu_2 (**e**), Cu_5 (**f**), Ag_0.5 (**g**), Ag_1 (**h**), Ag_2 (**i**), and Ag_5 (**j**) substrates.

Finally, the visualization of the MG-63 and SaoS-2 cells on the surface of the Ti substrate was performed using the scanning electron microscopy along with the cell morphology analysis (Figure 3).

The cells cultured on the unmodified and modified Ti surfaces were flattened and well-adapted, which indicated their viability. Intercellular junction characteristics for osteoblasts were clearly visible and the number of proliferating cells evidently decreased for higher concentrations of particular metals. These data stay in agreement with the increased toxicity observed for the growing metals concentration, as demonstrated by the cytotoxic assays (vide supra, Figure 1 and Figure 2).

To sum, the cell adhesion, growth, and proliferation levels clearly proved no significant cytotoxicity of the majority of investigated materials. Both the topography and the chemical properties of the substrate surface ensured beneficial effects for the proper culturing of osteoblasts in vitro. This confirms that the proposed surface modifications of the Ti substrates, in particular Zn_2, Zn_5, Cu_2, and Ag_0.5, should allow facile osseointegration.

**Figure 4:**
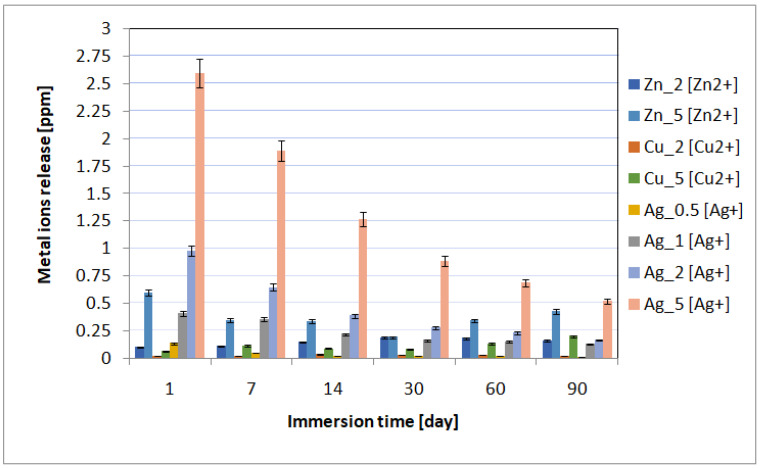
The kinetics of the metal ion release during the UHQ water incubation up to 90 days.

The spectrometric analysis showed that the metal ion release depended on the immersion time and the concentration of incorporated nanoparticles (Figure 4). The highest metal ion release was observed on incubation day 1, and then it gradually decreased. The highest metal ion concentration in the biological environment was observed for the materials containing 5% of metal nanoparticles in the layers. Moreover, for individual layers, after the first day of incubation, the content of individual metals was 0.60 ppm for Zn_5, 0.06 ppm for Cu_5, and 2.6 ppm for Ag_5. The higher content of metallic ions released into the environment correlated with the antimicrobial tests results. The best antimicrobial effectiveness was achieved for the layers containing 5% silver nanoparticles. It is also worth noting that after 90 days of sample incubation, the release continued and the metallic ion content was recorded at the level of ≤0.5 ppm. The ICP-MS research revealed that the coatings also prevented disadvantageous release of elements (e.g., vanadium) from the substrate into the surrounding environment.

### 2.2. In Vivo Study

**Figure 5:**
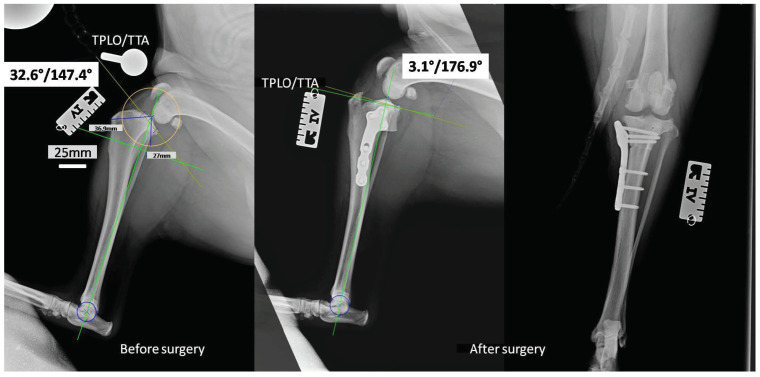
The X-rays of the stifle joint obtained for a representative dog before (horizontal) and immediately after the dog TPLO of a ruptured CrCL (horizontal and vertical).

Taking into consideration the results collected from the bactericidal effectiveness and cell viability studies, we decided to use the implants covered with layers containing 0.5% AgNPs for in vivo studies on canine patients, the surgery using the TPLO implants modified with the layers containing silver nanoparticles. The in vivo studies were successful. None of the operated-on dogs revealed deviations in the postoperative period, and all the dogs returned to full fitness. In the X-ray pictures taken before and after the operation, the tibial inclination angle and tibial plateau angle (TPA) were below 5°, with an average of 3.1° (Figure 5). Such parameters complied with the general treatment standards and ensured the correct position of the plate and bolts. They represented the optimal angle of tibial plateau rotation providing joint stability in cranial cruciate ligament-deficient stifles [29].

**Figure 6:**
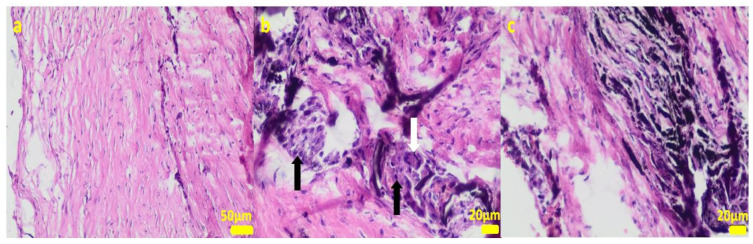
Sample 1:fibrous connective tissue (**a**) with small foci of inflammatory infiltrates consisting of macrophages (black arrows) including multinucleated giant cells (white arrow; (**b**)) and accumulation of fine fibrillar material and black pigment (**c**). Hematoxylin-eosin staining, magnification 400× (**b**,**c**), 200× (**a**).

**Figure 7:**
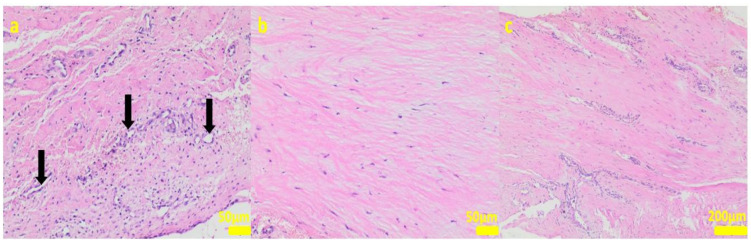
Sample 2:at the sample periphery, the fibrous connective tissue abundant in cells and small blood vessels (black arrows, (**a**)) with areas of maturation to compact fibrous tissue (**b**,**c**). Hematoxylin-eosin staining, magnification 200× (**a**,**b**), 40× (**c**).

**Figure 8:**
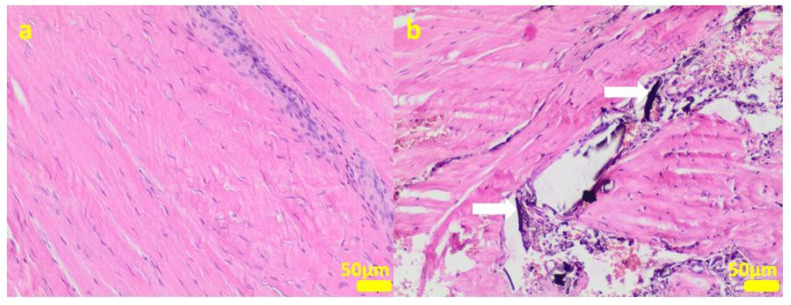
Sample 3:the compact fibrous connective tissue (**a**). Sample 4:the compact fibrous connective tissue with fine fibrillar material and black pigment (white arrow; (**b**)). Hematoxylin-eosin staining, magnification 100×.

**Figure 9:**
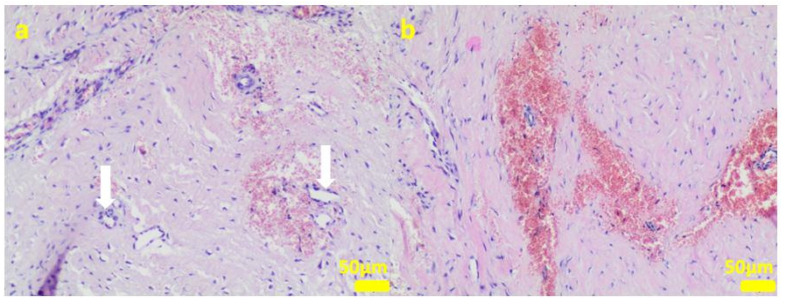
Sample 5:at the sample periphery, the fibrous connective tissue abundant in small blood vessels (white arrows) with areas of maturation to compact fibrous tissue (**a**), and multifocally hemorrhages (**b**). Hematoxylin-eosin staining, magnification 100×.

**Figure 10:**
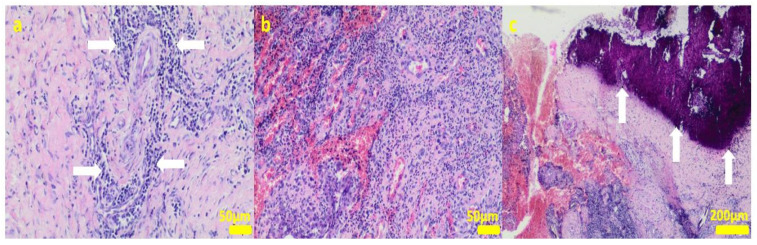
Sample 6:the compact fibrous connective tissue with perivascular inflammatory infiltrate consisting mainly of plasma cells (white arrows; (**a**)) with focal area of granulation tissue abundant in small blood vessels with mixed inflammatory infiltrate (**b**) and area of calcification (white arrows (**c**)). Hematoxylin-eosin staining, magnification 100× (**a**,**b**), 40× (**c**).

During the removal procedure, the implant proved to be very well-adhered to the tissue. The microscopic examinations of the collected samples (all the tissues collected after 3 months of implantations from the implant/bone contact area; each sample from the different patient) did not reveal microorganisms. Moreover, there was no inflammatory response suggesting a bacterial infection (Figure 6, Figure 7, Figure 8, Figure 9 and Figure 10). Although inflammatory infiltrates were found, they were more typical for a foreign body reaction (granulomatous inflammation in sample 1) or a chronic antigenic stimulation (plasmocytic infiltrate in sample 6).

The microscopic lesions observed in the examined samples suggested the correct tissue reparation process, starting from the granulation tissue up to the mature compact fibrous tissue that is typical for postsurgical scars. The implant, i.e., the TPLO plate and screws covered in the hybrid layer containing silver nanoparticles, seemed to facilitate the healing process; therefore, some features of granulomatous inflammation and plasmocytic perivascular infiltrates were present in the examined samples. Generally, both such microscopic findings and soft tissue calcification processes are observed in a natural healing process.

In all the canine patients, the diagnostic tests of the swabs collected from the tibia wounds did not reveal the anaerobic and aerobic bacteria growth in the cultures (negative result).

## 3. Discussion

In general, G (+) bacteria are more sensitive to the action of ZnONPs in comparison with G (−) [30]. However, the literature data regarding the metallic nanoparticles’ activity show some variations. The results depend on the nanoparticles’ size (the smaller the nanoparticles, the higher the biological activity), on the synthesis method, and directly on the test used to determine this activity. For instance, the 50–60 nm ZnONPs in the case of G (+) *S. aureus* bacteria acted once at the level of 100 µg/mL [31], and in another case, the 50 nm ZnONPs were bactericidal at 5000 µg/mL [32]. Raj et al. [33] showed that 25 µg/mL of zinc oxide nanoparticles had a bactericidal effect on *E. coli*, but Ali et al. [34] showed that these microorganisms were affected by the 2400 µg/mL dose. In the case of G (−) *P. aeruginosa* bacteria, there are also reports that a dose of 50 µg/mL [35] or 2800 µg/mL of ZnONPs is bactericidal (Ali et al. 2016). The bactericidal activity of ZnONPs consists of inter alia, disturbing the membrane potential of bacterial cells and disrupting cell membranes [36].

Copper nanoparticles interact with the microorganism cell wall, they affect the DNA synthesis in bacterial cells, and are responsible for the production of hydroxyl radicals that damage proteins [37]. CuNPs synthesized by the team of Ruparelia et al. [38] had a bactericidal effect on *E. coli*, depending on the tested bacterial strain in the dose from 160 to 300 μg/mL.CuNPs also inhibit the growth of three strains of *C. albicans* (the minimum concentration inhibiting the growth of the fungus was 129.7 μg/mL). Additionally, the authors observed the cell wall being disrupted by CuNPs and the leakage of the cytoplasmic content [39,40]. In another work, Sampath et al. [41] demonstrated the bactericidal effectiveness of Cu nanotubes against *S. aureus* at the 50 µg/mL dose. Silver nanoparticles, on the other hand, have the strongest bactericidal effect, which was also confirmed by our research. In general, the antibacterial activity of AgNPs against Gram (−) bacteria is stronger than against the Gram (+) ones. This phenomenon can be explained by different thicknesses of the cell walls of Gram (+) (30 nm) and Gram (−) (3–4 nm) bacteria, consisting mainly of peptidoglycan [42]. In experimental studies, Panpaliya et al. [43] demonstrated that AgNPs solution exhibited a higher bacteriostatic and bactericidal effect against five oral pathogenic bacteria than chlorhexidine. The mean minimum inhibitory concentrations (MICs) of AgNPs ranged from 2.82 ± 0.68 μg/mL to 90 ± 22.36 μg/mL, while the MIC of chlorhexidinegluconate was significantly higher (between 150 ± 55.90 μg/mL and 450 ± 111.8 μg/mL). Additionally, the biocidal and cytotoxic properties of AgNPs depend on their linear size and shape, as well as the concentration of the nanoparticles themselves [44]. We observed the following pattern: the higher the concentration of metallic nanoparticles in the particular AgNPs in the hybrid layer, the stronger the antimicrobial and thus cytotoxic effect on cells. Similar results were reported in the literature by Begum et al. [45]. Skomorokhova et al. [46] observed the effect of the AgNPs nanoparticles’ size on the antibacterial effectiveness, the smaller the nanoparticles, the stronger the dose-dependent and exposure time-dependent antibacterial effect. All AgNPs inhibited the metabolic activity of *E. coli* cells in line with the increasing concentration and the action duration, but no correlation between the cytotoxicity level and the nanoparticle size was found. In our research, the results clearly showed that the nanoparticles’ size had a major influence on the biocidal activity against Gram (+) and Gram (−) bacteria, which correlated with the cytotoxic activity against osteoblastic cells. The nanoparticles’ size in the individual layers was: 20–140 nm for ZnNPs, 100–500 nm for CuNPs, and 5–20 nm for AgNPs. Moreover, we obtained a spherical shape only in the case of AgNPs [25]. The strong biocidal activity of AgNPs also correlated with the ion release findings. The higher the concentration of nanoparticles introduced into the layer, the higher the released active ion concentration was. Wang et al. [47] observed a stronger bactericidal effect of AgNPs in the composite based on PVA than in the chitosan-based, which correlated with a higher concentration of silver ions released from the PVA material. In general, the active metal ion release has a major influence on the cytotoxicity of the tested materials. Our research showed that the highest active ion concentration was observed for the layers containing the highest proportion of metallic nanoparticles. In addition, a relatively high content of active ions was noted for the silver-enhanced samples (particles of the smallest size and spherical shape). Therefore, the smaller the nanoparticles’ size and the higher the released ions’ number, the higher the probability of cytotoxic activity. Xie et al. [48] observed a significantly reduced viability of MG63 osteoblast cells exposed to AgNPs at the 10 and 20 μg/mL concentrations, regardless of the exposure duration (24, 48 or 72 h). The size of the nanoparticle agglomerates in the cell medium was 201 nm, approx. 4 times the size of the original AgNPs (approx. 60 nm). In contrast, the exposure to low doses of AgNPs (0.5 and 5 µg/mL) for 24 h and 48 h stimulated the cell growth. However, after 72 h, the authors observed a dramatic decrease in the cell proliferation rate, except for the 0.5 µg/mL dose materials whose toxicity was significantly lower.

While accumulating in the cell, smaller AgNPs result in cytotoxicity, change the endothelial cell monolayers morphology, and promote cytokine release. The AgNPs measuring 5–28 nm can produce greater amounts of hydrogen peroxide and induce higher inflammasome formation since they cause stronger cathepsins leakage from lysosomes, induce more efflux of intracellular K+, and produce more superoxide in mitochondrial membranes [49]. Stoehr et al. [50] described how the silver nanoparticle’s shape influenced cytotoxicity. Namely, silver wires strongly affected the alveolar epithelial cells, whereas spherical silver particles had no effect. Moreover, AgNPs may act differently on different cells. For instance, neither lung fibroblast cells (IMR-90) nor glioblastoma cells (U251) exposed to a low dose of AgNPs revealed cytotoxicity but the cell proliferation was inhibited. Vergara-Llanos et al. [51], studying copper and zinc nanoparticles, observed the strain-dependent antibacterial activity of CuNPs and ZnONPs in a monospecies bacterial mode. They also noticed that ZnONPs, at a concentration of 150 μg/mL, caused toxicity in human gingival fibroblasts (HFGs). In the case of CuNPs, this phenomenon occurred at a concentration of 100 μg/mL.Pati et al. [52] revealed that after the oral exposure to mice, the use of higher doses of ZnONPs (70 μg/mL, a particle size between 200 and 250 μm) induced cytotoxicity in murine macrophages. The cell viability decreased because of the oxidative stress responses and the DNA damage in macrophages. Various literature data indicate that both the size of nanoparticles and their content have a strong influence on the cell viability and the appearance of cytotoxic effects.

In our study we wanted to find a proper balance between such a dose of metallic nanoparticles that would provide the strong biocidal activity and the lowest cytotoxic effect on cells. That is why we used the antimicrobial silver nanoparticles in the TPLO implants to minimize the risk of infection and improve the healing period of soft tissues. On the basis of our in vivo results, it was proven that 0.5% of AgNPs was safe for animals. Our work proved that faster tissue regeneration took place in comparison with pure TPLO implants. Our results stay in agreement with the recently published data revealing that silver nanoparticles facilitate the healing process of rat muscle tissue. In the literature, AgNPs not only had a bactericidal effect but also stimulated the osteoblast proliferation, promoting better osseointegration [53,54,55,56]. Kumar et al. [57] investigated the wound-healing process of albino rats treated with cream formulations with different AgNPs contents. The authors noticed that at the highest silver content percentage, the wound area was reduced, the collagen deposition increased, and more fibroblasts appeared with fewer macrophages and tissue edemas.

In summary, our results demonstrated the strong biocidal properties of hybrid layers with low-dose AgNPs against Gram-positive and Gram-negative bacteria without cytotoxic effects on the MG-63 and Saos cells. Moreover, AgNPs exert a beneficial effect on the tissue healing process and faster regeneration, suppressing postoperative wound inflammation.

## 4. Materials and Methods

### 4.1. Material Manufacturing 

**Figure 11:**
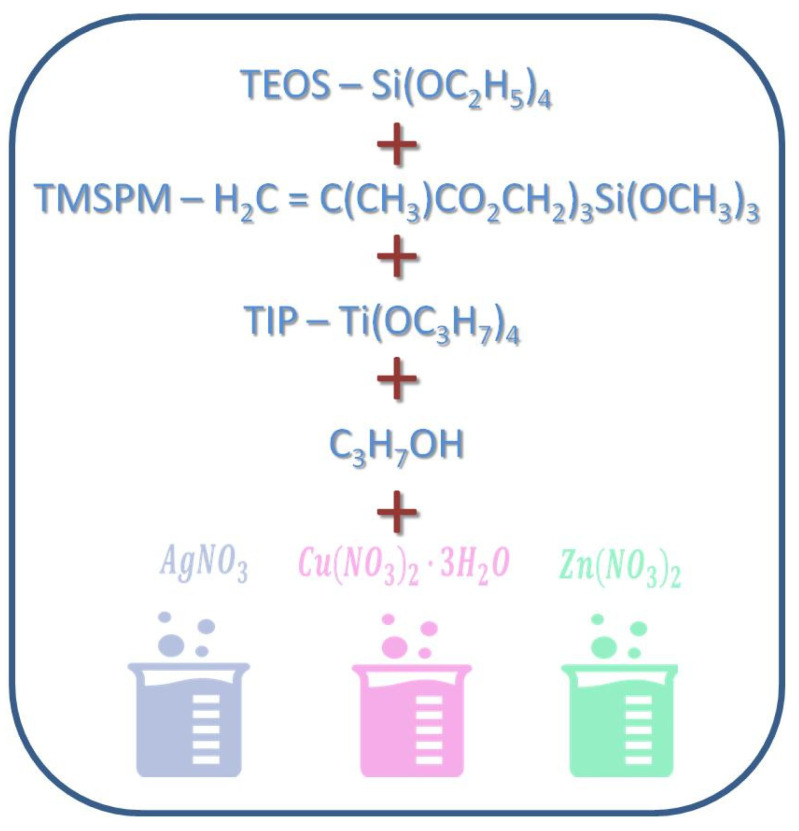
Scheme of hybrid coatings obtaining.

Metallic substrates (discs of a 1 cm diameter) and TPLO plates and screws fabricated of the TiAlV alloy were manufactured by Medgal (Księżno, Poland). Composite hybrid (organic–inorganic silicate) sols for coating their surface were prepared by the sol-gel method (Figure 11) according to Ziabka, et al. [25]. The chemical compositions of disc layers were designed with the 0.5, 1, 2, and 5 mol % concentrations of silver precursors and with the 2 and 5 mol % concentrations of copper and zinc precursors, respectively. Lower contents of silver nanoparticles (0.5 and 1%) were tested due to the strongest effect of AgNPs against Gram+ and Gram- bacteria as well as the potential cytotoxic effects of higher silver concentration. The chemical composition of the layers applied to the TPLO system contained 0.5 mol % concentration of silver precursors. The dip-coating technique was used to cover the metallic samples and the TPLO implants with hybrid layers. The conditions of the deposition process were as follows: immersion speed: 50 mm/min; drying in ambient conditions: 24 h; thermal treatment: 80 °C/10 min and 130 °C/15 min. Detailed characterization of coatings structure as well as physicochemical properties were presented in our previous paper (Ziabka, et al. [25]). In this study, the nomenclature of the samples was used as presented in Table 2. 

### 4.2. Methods of Examinations

#### In Vitro Bactericidal Efficacy Tests

A modified method according to the ASTM E 2180–07 norm “Standard Test Method for Determining the Activity of Incorporated Antimicrobial Agent(s) in Polymeric or Hydrophobic Materials” [58] was used to study the antimicrobial activity of discs: TiAlV, TiAlV/h and TiAlV/h containing Ag_0.5, Ag_1, Ag_2, Ag_5, Cu_2, Cu_5, Zn_2, and Zn_5 (3 of each group). The method was described in detail by Ziabka et al. (2020) [59]. The bacterial/fungi suspensions (1.5 × 10^5^ colony-forming unit (CFU)/mL) were prepared for bacteria: *Staphylococcus aureus* ATCC 25923G (+), *Enterococcus faecalis* ATCC 19433G (+); bacteria: *Escherichia coli* ATTC 25922 G (−), *Pseudomonas aeruginosa* ATCC 27853 G (−); and fungus: *Candida albicans* ATCC 90028. Next, 1 mL of the bacterial/fungal suspension (separately for each bacteria/fungi species) was added to the 100 mL solution consisting of 0.3% agar solution and 0.85% NaCl (soft-top agar). In 6-well cell culture plate (wells of 3.5 cm in diameter), 3 discs of the same type were laid out and covered with 50 μL of soft-agar containing bacteria or fungi. One type of bacteria/fungi was applied into one well. The plates were then incubated at 37 °C for 24 h and then the samples were transferred to 5 mL test tubes containing 2 mL of BHI broth (Brain Heart Infusion). After 1min sonication of the samples and 30minvertexing at 37 °C, 400 μL of the BHI-bacteria/fungi suspension was mixed with 600 μL of straight BHI. Next, 100 μL of the sample was planted on the BHI agar medium and incubated for 24 h at 37 °C. After that period, the colonies were counted.

### 4.3. Cell Cultures

The osteosarcoma cell lines (MG-63 and Saos-2) were purchased from ATCC (Manassas, VA, USA) and cultivated according to the manufacturer’s recommendation in Dulbecco’s Modified Eagle Medium (DMEM, Corning) and McCoy’s 5A (modified) Medium (Biological Industries) containing 10% fetal bovine serum (FBS), respectively. The media were supplemented with 1% penicillin/streptomycin. The cells were cultured on standard polystyrene plates (NEST Biotechnology) under controlled atmospheric conditions (37 °C temperature, 5% CO_2_). The culture medium was changed every 2–3 days. T the cells were sub-cultured after 3–4 days when the confluence reached about 80%. For passaging, the cells were detached with trypsin/EDTA and subsequently seeded. The same density of seeded cells was used in all the experimental approaches. The cells were seeded at 25 × 10^3^ cells/mL (25,000 cells per disk in a 24-well plate) in a serum-free medium and incubated at 37 °C for an appropriate period of time. Prior to use, the substrates were sterilized by triple soaking in pure ethanol and then triple rinsing in sterile PBS (Corning Life Science and ALAB, Warsaw, Poland). Every 2 days the medium was replaced with a fresh one.

### 4.4. Cell Viability Assay

In order to compare the modified surfaces regarding the cell attachment efficiency, the cytotoxicity assay was performed to establish the cell viability and growth levels. The Alamar Blue assay was carried out after 3 and 6 days of cell incubation on the investigated surfaces. The cells were incubated for 2 h with resazurin sodium salt (50 mg/mL in PBS) at 37 °C in the dark. The fluorescent response was measured at 605 nm (560 nm excitation wavelength) using a microplate reader (Infinite 200M PRO NanoQuant, Tecan, Männedorf, Switzerland). The cytotoxicity value was calculated as a relation between the percentage of living cells on the examined substrates (S) and the percentage of living cells on a pristine polystyrene plate (S_0_). All tests were repeated at least 3 times and the results are presented as a mean value + standard deviation (SD).

### 4.5. Cell Death Assay via Flow Cytometry

As a complementary analysis to the cytotoxicity test, the flow cytometry (FACS) was employed to assess the proper cell proliferation. Viable, early/late apoptotic, and necrotic cells were analyzed using the PI/Annexin-V-FITC (Thermo Fisher Scientific Inc., Waltham, MA, USA)double staining assay after 6 days of cell growth and proliferation on the studied substrates. The cells were washed with PBS, harvested with trypsin, then stained with 2 μL of Annexin-V-FITC and 2 μL of PI (50 μg/mL). The mixture was incubated for 30 min in the dark at RT. The cells were analyzed using the BD Accuri C6 Flow Cytometer (Bio-Rad, Hercules, CA, USA) with emission filters of 515−545 nm for FITC (green) and 600 nm for PI (red). A total event of 10,000 cells per sample was acquired. The experiments were repeated at least twice for each tested sample.

### 4.6. Cell Attachment and Growth Assay via Scanning Electron Microscopy (SEM)

The Nova NanoSEM 200 scanning electron microscope (FEI, Eindhoven, The Netherlands) was used to visualize the cell attachment and growth on the investigated surfaces. At the end of the 5th day, the MG-63 and Saos-2 cells were fixed with 2% glutaraldehyde (Sigma-Aldrich, Poznań, Poland) and dehydrated with graded ethanol (ranging from 20% to 100% (*v*/*v*)). Next, the samples were air-dried and visualized. The observations took place in low vacuum conditions, using the Low Vacuum Detector (SE) with the accelerated voltage of 10–18 kV. 

### 4.7. Ion Concentration Release Test

The TiAlV substrates and the hybrid layers covering TiAlV substrates were incubated in 30 mL of UHQ water at 37 °C for 3 months. The in vitro release of silver, copper, and zinc ions was studied by means of the inductively coupled plasma mass spectrometry (ICP-MS), using the ICP-MS Perkin-Elmer Plasma 6100 spectrometer (Perkin-Elmer, Waltham, MA, USA). Prior to performing the ICP-MS analysis, the filtered samples were acidified with nitric acid, up to the final concentration of 0.1 mol/L, in order to prohibit the silver ion (Ag^+^) reduction into metallic silver. The silver, copper, and zinc concentration values of the investigated samples were determined using the ICP-MS at *m*/*z* 107 (Ag), 63 (Cu), and 65 (Zn), applying the external standard calibration procedure.

### 4.8. Tibial Plateau Leveling Osteotomy (TPLO)

Six dogs with clinical signs of the damaged cranial cruciate ligament were enrolled in the study having obtained their owners’ written consents. All the canine patients enrolled for the study revealed positive compression tests with signs of lameness. The mean age of the dogs was 43 months, the mean weight was 32.7 kg. Before the surgery, the patients underwent basic blood tests (complete blood count + biochemistry) and the unstable knee joints were X-rayed in a lateral projection to assess the degenerative changes, determine the tibia angle, plan osteotomy and finally, select appropriate implants. Each patient was anesthetized according to the following schedule. For premedication, a mixture of 0.005 mg/kg dexmedetomidine, 0.3 mg/kg methadone, and 0.1 mg/kg midazolam was administered intramuscularly in 1 syringe. After about 15 min, a cannula was inserted, and the 50 mg/kg antibiotic Ceftriaxone was administered intravenously. In the meantime, the operated limb was shaved on one side and then the dog was placed in sternal recumbency with pelvic limbs extended cranially. The mixture of preservative-free 0.5% ropivacaine (0.5 mg/kg) with preservative-free 0.1% morphine (0.1 mg/kg) was administered into the epidural space (between the seventh lumbar and first sacral vertebrae). In total, 1 mL of the mixture was administered per 5 kg of body weight. The dog laid for 20 min on the operated limb so that the administered drugs could anesthetize the relevant nerves (in the meantime, the other side of the limb was shaved and X-rayed). For anesthesia induction, propofol was administered intravenously at a dose of 1–2 mg/kg, and then the dog was connected to the inhalation anesthesia machine (isoflurane). Before the procedure, the 0.2 mg/kg meloxicam dose was administered subcutaneously and the antibiotic Lincomycin with Spectinomycin intramuscularly. During the operation, the 10 mL/kg/hour crystalloids were administered intravenously and vital parameters (ECG, SpO2, EtCO2, BP, and temperature) were continuously monitored. Having prepared the surgical field in a routine manner, the knee joint mini arthrotomy was performed to assess the menisci and confirm the damage to the anterior cruciate ligament. In all patients, the cranial cruciate ligament was completely ruptured. In the case of the damaged medial meniscus, the meniscectomy of the posterior part of the meniscus was performed. In the next stage, the tibia osteotomy was performed. After the appropriate displacement of the cut epiphysis, the developed implants were used to stabilize the joint. In the study we used a system of compression-lock plates for TPLO with 3.5 mm-diameter screws covered with the hybrid layers containing 0.5% AgNPs. The mean duration of the surgical procedure was 43 min. After the surgery, a single intravenous bolus of dexmedetomidine was administered prior to the control X-ray. The pictures were taken in 2 projections in order to evaluate the procedure and to assess the bone fusion before removing the implants. After the surgery, the patients were not treated with protective antibiotics; for 7 days they were administered analgesics (Meloxicam at a dose of 0.1 mg/kg body weight, Meloxidyl from CEVA, Warsaw, Poland). A soft dressing was applied on the wound for 4–5 days. In the postoperative period, the follow-ups were carried out in days 10–14, and then in weeks 6th and 12th. Having confirmed the proper bone fusion via X-ray, the implants were removed 3 months after the surgery. During the implant removal, a fragment of the surface material was collected for histopathological and bacteriological examinations. Eventually, after the implant removal, one more control visit took place between days 10–14.

### 4.9. Radiological Examinations

The X-ray examinations of the operated knees were performed before prior to the stabilization, right after the TPLO surgery and 12 weeks after the operation. The photos were taken using a MAXIVET 400 HF stationary camera (Sigmed, Cisek, Poland), at an exposure of 70 kV and 6 mAs.

### 4.10. Bacteriological Examination

When removing the implants, a swab was taken from the wound near the tibia. The collected material was used to culture aerobic and anaerobic bacteria for further studies. 

### 4.11. Histopathological Examination

The connective tissue fragments in the vicinity of the implants were collected for the histopathological examination to evaluate the tissue response to the implants. The collected samples were fixed in the 4% buffered formaldehyde and processed via the standard protocols (dehydration, clearing, and paraffinization). The sections from each paraffin block were cut into 4 µm thick slides and stained with hematoxylin-eosin. The microscopic examination was performed by a veterinary pathologist (RS) using the light microscope Olympus BX43, (Olympus, Tokio, Japan).

### 4.12. The Animal’s Owner’s Informed Consent form for Conducting In Vivo Research

Conducting in vivo research on canine patients did not require approval of Ethical Commission, as they were carried out in accordance with the regulation of the Polish minister of health of 27 November 2008, which states: “… It may happen that there are no medical products necessary for veterinary treatment. In such cases, to rescue and treatment of the patient it is allowed to waive the rules of the usage of medicines already implemented to the market. The method of dealing with such cases is updated in 2001/82/EC Directive”.

Additionally, the Polish Veterinary Code of Ethics, Art. 22 point: 1–4, Warsaw 2009, claims:

1. The vet is free to choose the methods of diagnosis, treatment, and prevention if it does not oppose the general rules;
2. The vets use, in their workplace, scientifically recognized methods of diagnosis and toxicity;
3. The vet should limit their activities only to those necessary. This rule does not apply to experiments;
4. In the event of the application of new, unproven methods of treatment, the vet should inform the animals’ owner and gain their permission for the treatment.

The study was conducted in compliance with the guidelines outlined in the Poland Animal Welfare Act and in the European Directive 2010/63/EU for animal experiments and the Good Clinical Practice (Federation of Veterinarians of Europe, FVE, Code of Good Veterinary Practice, 2003).

All pet owners signed the informed consent form:
Statement: the owner/guardian of the animal consent to perform medical and veterinary treatmentOwner or Guardian Details
Surname and name:
Designation and ID number identity:
Address:
Telephone number or other contact:
Description of an animal with the name:
Species:
Race:
Sex:
Age:
Ointment:
Ointment:
I, the undersigned, after having received comprehensive and exhaustive information, including the possibility of asking the questions, hereby fully consciously and irrevocably consent to the application of the above-mentioned anesthesia and to perform the treatments listed above, including any other necessary treatment.I have been informed that the anesthesia and the procedures performed involve a risk to life and health of the animal.I declare that I have complied with the fasting recommended for the animal, and after the procedure I undertake to care for it in accordance with the guidelines given to me that are understandable to me.SignatureSignature(Animal owner/guardian)(Veterinarian)

## 5. Conclusions

To evaluate antimicrobial activity and biocompatibility of TPLO implants covered with hybrid layers, the in vitro testes involving evaluation of MG-63 and Saos cells viability, antimicrobial efficacy, the metallic ion release, in vivo implantation and histopathology examination were performed. Based on the conducted research, it was found that the hybrid layer containing AgNPs revealed the strongest antimicrobial properties against Gram (+) and Gram (−) bacteria and against pathogenic yeast, *C. albicans*. However, the concentration of AgNPs exceeding 1% may be cytotoxic and result in cell necrosis and apoptosis. Therefore, the TPLO system, with 0.5% AgNPs, was tested in vivo on six canine patients to evaluate the implant antibacterial activity. The clinical studies and 3-month follow-up observation of the patients after tibial osteotomy knee surgery allowed for the anastomosis assessment using the developed new implants and the influence of antibacterial layers on the tissue regeneration process around implants. The radiological evaluation carried out in the postoperative period confirmed the optimal angle of tibial plateau rotation providing joint stability in cranial cruciate ligament-deficient stifles. It was also found that the TPLO implants covered by layers modified with AgNPs provided good biocompatibility in vivo and facilitated the bone healing process. This process also reduced the recovery time due to the faster recovery of the limb functionality and bone healing with no antibiotics administration compared with patients treated with currently used titanium implants. Moreover, a valuable feature of the developed implants is the fact that the developed layers may protect them against corrosion and surgical scratches. Therefore, the developed implants can be successfully applied to perform tibial plateau leveling osteotomy surgery.

Thus, we proved that the developed hybrid layers with AgNPs fabricated via the sol-gel technology and a relatively simple technique of their application (dip coating) increased the functionality of commercially available implant materials.

Further studies will be focused on the in vivo evaluation of implants in dogs with a higher body weight and the comparison studies with a control group in which implants without layers will be implanted.

## Figures and Tables

**Table 1:** The antimicrobial efficacy of the investigated materials (colony-forming unit).

Sample	*Staphylococcus aureus* ATCC 25923	*Enterococcus faecalis* ATCC 19433	*Escherichia coli* ATCC 25922	*Pseudomonas aeruginosa* ATCC 27853	*Candida albicans* ATCC 90028
Ti	1.8 × 10^4^	8 × 10^3^	5.1 × 10^4^	4.2 × 10^6^	2.9 × 10^4^
hybrid	1.4 × 10^4^	4.5 × 10^3^	2.2 × 10^4^	1.5 × 10^6^	1.8 × 10^4^
Ag_0.5	5 × 10^1^	0	0	0	3.0 × 10^4^
Ag_1	0	0	0	0	1.3 × 10^4^
Ag_2	0	0	0	0	1.0 × 10^4^
Ag_5	0	0	0	0	1.6 × 10^3^
Cu_2	9.2 × 10^3^	0	9.1 × 10^3^	1.3 × 10^6^	1.7 × 10^4^
Cu_5	2.6 × 10^3^	0	0	1.7 × 10^2^	1.8 × 10^3^
Zn_2	1.1 × 10^4^	4.4 × 10^3^	4.8 × 10^4^	1.5 × 10^6^	5 × 10^3^
Zn_5	6 × 10^1^	4.3 × 10^3^	3.2 × 10^4^	1.4 × 10^6^	2.8 × 10^2^

**Table 2:** The samples nomenclature.

Sample Characteristic	Sample Nomenclature
Titanium alloy Ti-6Al-4V	Ti
Titanium alloy Ti-6Al-4V with a pure hybrid layer	Hybrid
Titanium alloy Ti-6Al-4V with a hybrid layer containing 0.5% of silver nanoparticles (AgNPs)	Ag_0.5
Titanium alloy Ti-6Al-4V with a hybrid layer containing 1% of silver nanoparticles (AgNPs)	Ag_1
Titanium alloy Ti-6Al-4V with a hybrid layer containing 2% of silver nanoparticles (AgNPs)	Ag_2
Titanium alloy Ti-6Al-4V with a hybrid layer containing 5% of silver nanoparticles (AgNPs)	Ag_5
Titanium alloy Ti-6Al-4V with a hybrid layer containing 2% of copper nanoparticles (CuNPs)	Cu_2
Titanium alloy Ti-6Al-4V with a hybrid layer containing 5% of copper nanoparticles (CuNPs)	Cu_5
Titanium alloy Ti-6Al-4V with a hybrid layer containing 2% of zinc nanoparticles (ZnNPs)	Zn_2
Titanium alloy Ti-6Al-4V with a hybrid layer containing 5% of zinc nanoparticles (ZnNPs)	Zn_5

## Data Availability

The data presented in this study are available on request from the corresponding author.
